# Applications of Nanomaterials in Microbial Fuel Cells: A Review

**DOI:** 10.3390/molecules27217483

**Published:** 2022-11-02

**Authors:** Nabil. K. Abd-Elrahman, Nuha Al-Harbi, Noor M. Basfer, Yas Al-Hadeethi, Ahmad Umar, Sheikh Akbar

**Affiliations:** 1 Soil Fertility and Microbiology Department, Desert Research Center, Cairo 11753, Egypt; 2 Department of Physics, Umm AL-Qura University, Makkah 24382, Saudi Arabia; 3 Department of Physics, Faculty of Science, King Abdulaziz University, Jeddah 21589, Saudi Arabia; 4 Department of Chemistry, Faculty of Science and Arts, and Promising Centre for Sensors and Electronic Devices (PCSED), Najran University, Najran 11001, Saudi Arabia; 5 Department of Materials Science and Engineering, The Ohio State University, Columbus, OH 43210, USA

**Keywords:** microbial fuel cells (MFCs), proton exchange membranes (PEM), nanomaterials, biocompatibility, biofilm, cathode reactions, oxygen reduction reactions (ORR), nanotechnology

## Abstract

Microbial fuel cells (MFCs) are an environmentally friendly technology and a source of renewable energy. It is used to generate electrical energy from organic waste using bacteria, which is an effective technology in wastewater treatment. The anode and the cathode electrodes and proton exchange membranes (PEM) are important components affecting the performance and operation of MFC. Conventional materials used in the manufacture of electrodes and membranes are insufficient to improve the efficiency of MFC. The use of nanomaterials in the manufacture of the anode had a prominent effect in improving the performance in terms of increasing the surface area, increasing the transfer of electrons from the anode to the cathode, biocompatibility, and biofilm formation and improving the oxidation reactions of organic waste using bacteria. The use of nanomaterials in the manufacture of the cathode also showed the improvement of cathode reactions or oxygen reduction reactions (ORR). The PEM has a prominent role in separating the anode and the cathode in the MFC, transferring protons from the anode chamber to the cathode chamber while preventing the transfer of oxygen. Nanomaterials have been used in the manufacture of membrane components, which led to improving the chemical and physical properties of the membranes and increasing the transfer rates of protons, thus improving the performance and efficiency of MFC in generating electrical energy and improving wastewater treatment.

## 1. Introduction

Microbial fuel cell (MFC) is an innovative and eco-friendly technology. It is classified as one of the ways to produce renewable energy using organic waste, as it is considered environmentally friendly. In MFCs, a chemical is converted energy to electrical energy using microbes [1]. The MFC consists of either one or two chambers separated by a membrane. In the case of two chambers of MFC, the anode chamber contains the positive electrode, the cathode chamber contains the negative electrode, and the proton exchange membrane (PEM) is used to separate the two chambers and allows the proton transfer from the anode to the cathode. Organic or chemical waste is placed in the anode chamber. Bacteria play an important and effective role in the analysis of organic compounds present in the anode chamber and produce electrons and protons. Electrons transfer from the anode to the cathode through an external circuit, while the protons transfer through the PEM to the cathode [2]. There are many factors that affect the performance and efficiency of MFC, such as the organic material used, bacteria (biocatalyst), internal resistance, properties of the electrode material, the membrane, and the concentration of ions [3,4,5].

The electrode materials (anode and cathode) are important in the performance and efficiency of MFC in terms of the type of electrode material, electrical conductivity, biocompatibility, surface area, and non-toxicity to bacteria, which affects the efficiency of the oxidation and reduction processes of organic matter, electron transfer, and electric power generation. In addition, the PEM is an important component in the efficient operation of the MFC in terms of the type of membrane material and the size of the membrane pores, which affects the process of transferring protons and reducing the transfer of oxygen to the cathode [5]. In MFC systems, anode performance is a critical component that can influence the level of power output that can be reached. In general, the surface qualities of the anode material used have a significant impact on the anode response. As a result, the development of new materials and designs for anode electrodes is crucial to increase the efficiency of this technology for practical use. One goal of employing nanomaterials for anode manufacturing is to improve electron transfer mechanisms between microorganisms that function as biocatalysts in the anode chamber and the material that forms the anode itself to improve power output. Nanostructured materials can be used to modify the surface of an anode electrode composed of different sorts of materials or as the base anode material [6].

At the cathode, the final electron acceptor (e.g., oxygen) combines with the protons to generate the final product (e.g., H_2_O). The cathode materials directly dominate the kinetics of the oxygen reduction reactions (ORR) in the cathode chamber. ORR is primarily determined by the surface area, electrical conductivity, chemical stability, and activity of the cathode materials, among other factors. As a result, the cathode materials used and their alterations are critical for the optimization and improvement of MFC performance [7].

**Figure 1:**
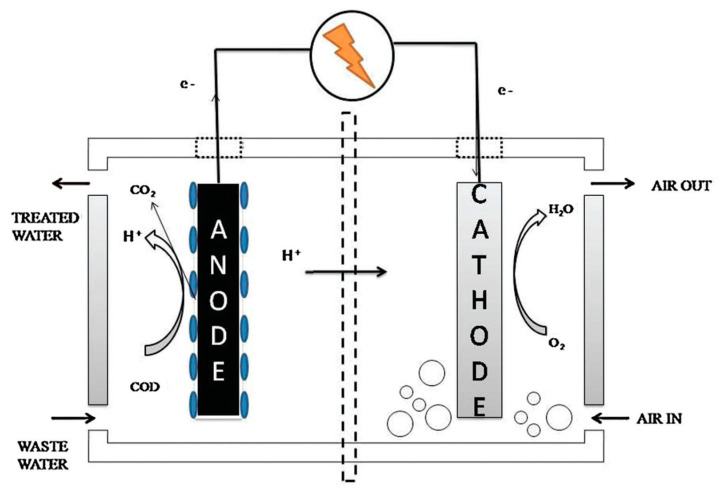
Schematic representation of a microbial fuel cell (MFC). Reprinted with permission from [11], Salva, N.; Anand, R.; Pandit, S.; Prasad, R. Utilization of Nanomaterials as Anode Modifiers for Improving Microbial Fuel Cells Performance. *J. Renew. Mater.* **2020**, *8*, 1581–1605. https://doi.org/10.32604/jrm.2020.011803. Copyright @ Tech Science Press (2022) under a Creative Commons Attribution 4.0 International License.

The anode and the cathode reactions of the MFC must be separated using the membrane that is fixed between the anode and the cathode chambers. The membrane acts as a physical conductor or separator, which facilitates the transfer of protons from the positive electrode to the negative electrode [8]. The membrane used in MFC must be characterized by low internal resistance, high proton conductivity, high energy recovery, and good chemical and physical stability, which helps prevent the transfer of oxygen from the cathode chamber to the anode chamber [9]. The current trend is to improve the performance of membranes and their physical and thermal properties by incorporating nanomaterials into membrane structures, which helps in achieving the desired properties and improving the performance of MFC [10]. Figure 1 exhibits schematic for the microbial fuel cell (MFC) [11].

The present review focuses on previous studies of nanotechnology applications and their impact on the performance and efficiency of MFC in energy production and wastewater treatment. There are three factors or basic parts in the installation and operation of the MFC. The first factor is the anode or the positive electrode, and the focus is on the use of nanomaterials that improve the performance and efficiency of the anode, such as increasing the surface area, increasing electron transfer, and the generation of electrical energy. The second factor is the cathode or the negative electrode, and the focus is on the use of nanomaterials that improve cathode reactions or ORR. The third factor is the PEM proton membrane and the use of materials and nanoparticles in the manufacture and installation of proton exchange membranes and their effect on proton transfer and preventing the transfer of oxygen from the cathode to the anode. The effect of anode, cathode, and membrane on improving the performance and efficiency of MFC in energy production and wastewater treatment is the focus.

## 2. The Anode

### 2.1. Properties of Anode Materials

In order to achieve appropriate results in terms of electrochemical efficiency, electron conversion, and bacterial adherence [12], the anode must have some of the following properties.

#### 2.1.1. Electrical Conductivity

Because the electrons released by bacteria are transferred to the anode, which then transfers to the cathode via the external circuit, conductivity is an important property of anode materials. Therefore, the anode material is responsible for allowing electrons to flow and increasing their speed [13]. Highly conductive materials aid in lowering bulk solution resistance and increasing electron transfer. To increase electron transfer, the interfacial impedance between the substrate and the electrode must be low [14]. Before constructing the anode for MFCs, the electrical conductivity of materials is frequently investigated.

#### 2.1.2. Surface Area

**Figure 2:**
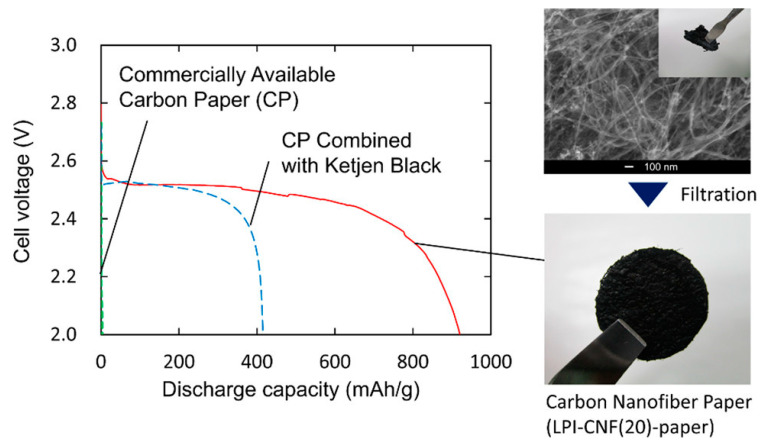
Discharge capacity of the prepared carbon nanofiber paper. Reprinted with permission from [15], Rambabu, G.; Bhat, S.D.; Figueiredo, F.M.L. Carbon Nanocomposite Membrane Electrolytes for Direct Methanol Fuel Cells—A Concise Review. *Nanomaterials* **2019**, *9*, 1292; https://doi.org/10.3390/nano9091292. Copyright @ MDPI (2022) under a Creative Commons Attribution 4.0 International License.

Anode surface area has a significant influence on the power output in MFCs, as demonstrated by Kazuki Sakai et al. [15]. Sakai et al. prepared carbon nanofibers with different diameters using a liquid pulse injection process and investigated how the length and diameters of the nanofibers affect their surface area, permeability, and electrical conductivity of the material [15]. Figure 2 exhibits the discharge curves of the prepared carbon nanofibers paper, which revealed high discharge capacity [15].

The resistance of the anode is directly related to the Ohmic losses of the fuel cell. In addition, it provides more active sites for bacterial growth and improves the electrode kinetics efficiency. Bacteria such as *E. coli*, *Geobacter* spp., and *Pseudomonas* spp. are efficiently and actively fixed to the surface of the anode, allowing efficient and direct electron transfer [16]. Since biological reactions take place on the surfaces of the anode, the surface area has a significant impact on the performance of the MFC [17].

#### 2.1.3. Biocompatibility

Biocompatibility of anode has significant importance in MFC processes because it is directly in contact with bacteria and their respiration process. Several materials, including copper, silver, and gold, are not deemed biocompatible for use as the anode in MFCs because they are prone to corrosion [18,19]. The toxicity of these materials can prevent microbial activity during MFC operation, resulting in decreased energy generation.

#### 2.1.4. Stability and Durability

Because of non-biocompatibility, chemical, and mechanical instability, long-term interaction of traditional anode electrodes with the substrate and inoculation of microorganisms in MFCs usually results in swelling. As a result, the physical stability of the anode is disrupted. Corrosion, thermal instability, and inadequate mechanical strength all contribute to swelling [19]. The anode surface should also be rough to separate water molecules and then provide additional active sites for bacteria adhesion [19].

#### 2.1.5. Ease of Access to Materials and Cost

Availability and cost of anode materials are critical considerations as they have a direct impact on the full cost of MFCs. Silver, gold, and platinum, for example, are very valuable and hard to come by. Metallic compounds and compounds derived from natural carbon can be an attractive alternative to expensive metals as anode materials in MFCs [20].

### 2.2. Materials Used in Anodes

Oxidation of the organic substrate, the electron transfer rate (ETR), and microbial adhesion are all performance parameters that can be affected by the anode used in MFCs. The quality and properties of the anode have a direct impact on energy generation [21]. Carbon, graphite, metal, metal oxides, and natural waste materials have been used as anodes in MFCs [16,22,23].

#### 2.2.1. Carbon Materials

Carbon materials are commonly used to fabricate anode because of their high conductivity, large surface area, low porosity, good biocompatibility, high chemical and thermal stability, ease of availability, and good electron transfer kinetics. Carbon materials were commonly used for anodes, such as carbon nanotubes (CNTs), carbon cloth, carbon paper, carbon rods, carbon meshes, carbon brushes, carbon felt, carbon fibers, and reticulated vitreous carbon [15,16,17,18,19,20,21,22,23,24,25]. Carbon cloth is the most used to fabricate the anode in MFCs. This type of carbon has high electrical conductivity, excellent flexibility, mechanical stability, and larger surface area compared to ordinary carbon paper, but it has the disadvantage of relatively high porosity due to large voids, high cost, and chemical instability that causes pollution and reduces the long-term stability of the anode [26].

Carbon paper is also used to fabricate the anode. Despite its high porosity, carbon paper is not recommended for use as an anode in MFCs due to its high cost [12]. Carbon brush is a very interesting carbon material that has a large surface area and good electrical conductivity through its bonding to titanium. They are widely used as anodes, but their disadvantage is the high cost [27]. Carbon mesh is commercially available at a low cost, but it has low electrical conductivity and poor mechanical stability, which leads to poor durability [23]. Carbon mesh as an anode in an MFC was investigated by Wang et al. [28] as a less expensive alternative to carbon cloth and carbon paper materials. To attain good MFC performance, they discovered that the carbon mesh needed to be pre-treated (heat, acetone, high temperature, or ammonia-gas process).

Carbon veil is a low-cost material, but it has moderate electrical conductivity and a highly porous structure that is utilized as an anode in MFCs to improve energy generation efficiency. The single-layer carbon veil is very brittle, but it is multifunctional and may be folded to create a 3D anode [29]. Carbon felt is one of the most widely used materials as an anode in MFC. It has good electrical conductivity, high porosity, and good mechanical stability, but its cost is relatively high [30].

Granular Activated Carbon (GAC) is a cost-effective and bio-compatible material, but it has low electrical conductivity and high porosity, which limits the flow of electrons and reduces electrochemical performance. This material will need to be modified further to improve its conductivity and decrease its porosity [31]. Borsje et al. [32] investigated three single carbon granules as bio-anodes in MFC to generate energy for two GACs with a large surface area and one graphite granule with a small surface area. They showed that GAC bio-anodes produced 1.3–2 times more energy than normal graphite bio-anodes. However, more research is required to identify the important variables that influence the granular system’s performance. Li et al. [33] investigated the use of GAC as an anode in an MFC and found that it generated 2.5 times more power density than carbon cloth as an anode. GAC demonstrated maximum columbic effectiveness as well as high energy output. These tests showed that the GAC’s increased surface area aided the growth of biofilm around the anode, enhancing the rate of organic substrate oxidation and improving overall energy generation.

#### 2.2.2. Graphite–Graphene Materials

Graphite, a type of carbon material, is often utilized as an anode material in MFCs [34]. Graphite is a two-species hybridized crystalline form of carbon atoms. Graphite’s potential advantages, such as biocompatibility, mechanical strength, and large surface area, make it an excellent material for the anode in MFCs. However, some disadvantages, including its high cost and limited conductivity, make it unsuitable for satisfying current commercial energy demands [35]. In general, graphite materials commonly used for the anode, such as graphite sheet or plate, paper, rod, granular, cloth, and brush, are more effective than using regular carbon as anode in MFCs.

Graphite flat and graphite rough as anode in MFCs were compared by Ter-Heijne et al. [36]. The graphite rough provided reasonable results in terms of energy generation and pollutant removal efficiency, according to their findings. Graphite felt was studied by Vijay et al. [37] as an anode in an MFC using nuclear wastewater sludge as an inoculum source in order to remove uranium. Uranium removal rates were about 32–90% for 24–120 h of MFC operation. Power output was relatively low due to a poor generation of electrons. Because the nuclear waste contained various unstable elements, the graphite felt failed to provide long-term stability for bacteria growth in that case.

**Figure 3:**
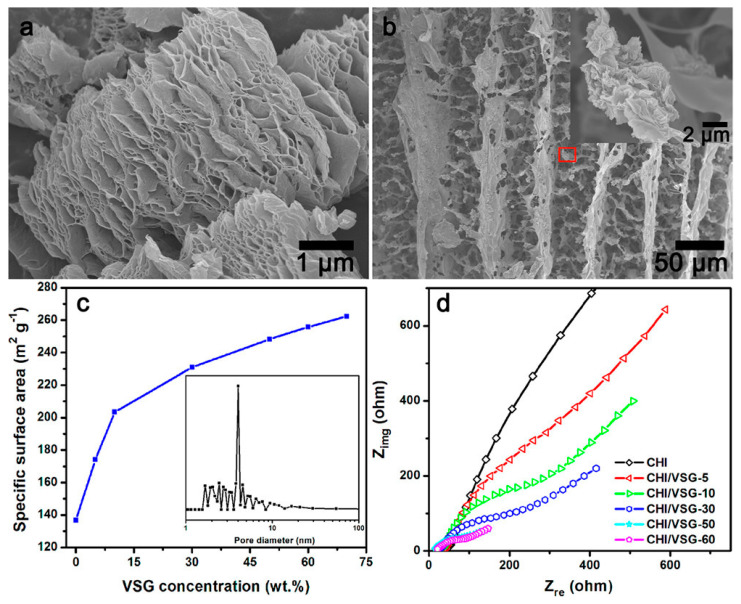
FESEM images of (**a**) vacuum-stripped graphene and (**b**) chitosan/VSG powder, (**c**) the plot of specific surface area vs. VSG concentration of the CHI/VSG scaffolds, and (**d**) Nyquist plots of different CHI/VSG electrodes. Reprinted with permission from [39], He, Z.; Liu, J.; Qiao, Y.; Li, C.M.; Tan, T.T.Y. Architecture Engineering of Hierarchically Porous Chitosan/Vacuum-Stripped Graphene Scaffold as Bioanode for High Performance Microbial Fuel Cell. *Nano Lett.* **2012**, *12*, 4738–4741. https://doi.org/10.1021/nl302175j. Copyright @ American Chemical Society (2022).

Graphene is one of the promising and emerging carbon materials that have aroused great interest as an anode in MFC, consisting of a single sheet of graphite with a two-dimensional crystalline (2D) structure. It has high electrical conductivity, good biocompatibility, excellent mechanical properties, chemical and thermal stability, greater diamagnetism, and nonlinearity than simple graphite [35]. Different studies reported the use of graphene and its derivatives as anode in MFCs [38,39]. He et al. prepared a hierarchically porous chitosan/vacuum-stripped graphene scaffold as a bioanode for high-performance microbial fuel cells (Figure 3) [39]. He et al. reported many chitosan/vacuum-stripped graphene (CHI/VSG) structures by varying the VSG concentration. As the concentration of VSG increased, the specific surface area increased as well [39]. The CHI/VSG scaffolds, however, were susceptible to breaking when the VSG concentration approached 70% by weight. The ideal VSG concentration was 50 weight percent (CHI/VSG-50), with a noteworthy maximum power density of 1530 mW/m^2^, which was 78 times greater than the value of carbon cloth anodes (19.5 mW/m^2^) [39]. Figure 3 exhibits the typical FESEM images for VSG and CHI/VSG scaffolds, their specific surface area, and Nyquist plots of different CHI/VSG electrodes.

Chen et al. [40] fabricated a three-dimensional (3D) graphene sponge to be used as an anode in MFC. They showed that a higher power density than that obtained with carbon felt was attained, probably due to the microporous structure of the graphene sponge allowing a better growth of the bacteria, resulting in a higher MFC performance.

#### 2.2.3. Metal/Metal Oxides Materials

Metal/metal oxides such as silver, nickel, gold, aluminum, copper, stainless steel, molybdenum, iron, and titanium were used as anodes in MFCs. They performed well, but their durability and biocompatibility were not suitable for bacteria adhesion [41]. Some of these materials are discussed below.

##### Molybdenum

Yamashita and Yokoyama [42] used a molybdenum-based anode in an MFC to reach a power density of 1296 mW/m^2^, but it had corrosion issues in long-term operation. Furthermore, metal oxides’ dimensional properties, such as vertical 3D porous-based titanium oxide nano-sheets, provided an active surface area that aided electron transport and molecular diffusion. When metal oxides were used as anodes, they achieved low bacterial adhesion and low electron transfer through bacterial respiration [43].

##### Titanium

Using titanium rod as an anode for the treatment of vegetable oil-based industrial wastewater in MFCs, Firdous et al. [44] achieved a maximum voltage output (5839 mV), but the long-term stability of this material remains a challenge. Metals are commonly considered highly conductive materials because their atoms form a matrix where the outer electrons move freely [45]. Bacterial adhesion to metal surfaces is typically much less effective than on other surfaces, such as carbon materials [46].

##### Stainless Steel

You et al. [47] used stainless steel meshes modified by gas diffusion treatment as anodes in an MFC to achieve a power density of 951.6 m W/m^2^. According to Santoro et al. [26], most metals used as anode in MFCs, such as gold, silver, titanium, and platinum, were only used for short periods of time due to corrosion issues and the high cost of noble metals such as gold and silver [48].

#### 2.2.4. Natural Waste Materials

Fabrication of anode from natural materials such as biomass wastes has sparked a lot of attention recently because of various benefits such as the use of recyclable resources, material availability, and material stability [49]. Natural waste-derived materials are the most efficient materials for fabricating anode for MFCs because they are less expensive than traditional materials and have all the fundamental features of ideal anode material. The micro/mesoporous 3D structure of natural waste-derived materials showed a high rate of electron transfer and good electrokinetics mechanism for electrochemical processes in MFCs [50]. Spongy 3D anode for MFCs used natural waste materials were fabricated as low-cost and high-performance materials [51]. Natural waste materials commonly available and used for anode fabrication are almond shells, forestry residues, wastepaper, wood-derived wastes, corn straw, chestnut shells, and silk cocoons [52]. On the other hand, there are several waste materials that have not been tested for anode fabrication yet. Furthermore, none of the previously produced anodes made from natural wastes had a conductivity efficiency high enough to meet the present energy demand for MFCs.

### 2.3. Nanomaterials as Anode in MFCs

Commonly used anode nanomaterials include carbon materials, graphene metal, and metal oxides have good conductivity, high surface areas, good chemical stability, and good biocompatibility. This final attribute is critical because successful electron transfer requires an affinity relationship between the microorganisms and the electrode. Carbon materials are among the most often utilized materials for MFC anode manufacturing since they are of low cost. Carbon cloth, carbon fiber, and carbon felt (or brush) are commonly used as anodes in MFCs.

#### 2.3.1. Carbon Nanomaterials

Carbon-based nanomaterials are usually used to raise the efficiency of the anode in MFC cells because carbon nanomaterials are characterized by an increase in the electrical conductivity efficiency and an increase in the surface area of the electrode, which provides many active sites that are useful in the binding of bacteria and thus increases the electron transfer rates [53].

##### Carbon Nanotubes (CNTs)

CNTs have been used in the modified anode electrodes in MFC. It was found that carbon nanotubes showed good results in the modifications that were added to the anode because they provide a large surface area and high electrical conductivity. They also resulted in a higher power density than the unmodified anode due to the increase in the number of active sites on which bacteria grow and thus result in an increase in electron transfer rates. CNTs are characterized by their specific fibrous structure, high toughness, high mechanical strength, large surface area, and good thermal stability [54]. Roh et al. [55] modified carbon paper nanotubes as an anode. As a result, the internal resistance of the anode was reduced to 258 Ω (CNTs + carbon paper), which was 1163 Ω in carbon paper before modification. Additionally, the highest energy density increased to 290 m W/m^2^ from 241 m W/m^2^. This is due to the increased anode surface area caused by the addition of CNTs. CNTs have a large specific surface area, which makes them ideal for electroactive bacteria attachment and growth. It improves the rate of electron transfer from bacteria to the anode. However, CNTs have been reported to have cytotoxicity, which can inhibit cell proliferation and cause cell death [27]. Therefore, the dosage of CNTs, when used for anode modification, must be strictly controlled.

##### Carbon Nanofibers (NFs) and Nanospheres (CNs)

Natural and recyclable materials, such as organic wastes and glucose, are commonly employed as raw materials in the production of high-performance carbon nanofibers [56] and nanospheres. The organic matters are converted into a solid phase to form uniform sizes, a better appearance, and a higher surface area of nano-spheres through high-temperature carbonization and activation. Additionally, the as-prepared nano-sphere shows good biocompatibility and electrical conductivity in long-term operation [57].

Carbon nano-spheres showed an increase in the active sites for bacterial adhesion, which led to an increase in electron transfer rates and a significant improvement in energy density and non-toxicity to bacteria, which indicates the possibility of using them in practical application, as they are low cost [58,59].

##### Graphene Nanomaterials

Graphene is the basic unit of other graphite materials. It is a 2D composed of hexagonal carbon. It has good electrical conductivity, a large surface area, good stability, and good biocompatibility. Zhang et al. [60] employed graphene to improve stainless steel mesh and achieved a maximum power density of 2668 m W/m^2^, which was 18 times higher than the stainless-steel mesh used as an anode in MFC. Graphene was suitable for bacteria enrichment, which facilitated electron transfer from the bacteria to the anode.

##### GAC Nanomaterials

GAC, with a porous structure, which is made from charcoal, various fruit shells, and high-quality coal as raw materials, is frequently used for organic adsorption. GAC has a lower fabrication cost than graphene and diamond nanoscale materials, and its large accessible surface area is excellent for improving anodic performance [61]. It has many forms, such as powder, granular, fiber, and cellular shapes. According to Chen et al. [62], activated microporous-mesoporous carbon prepared with chestnut shell as anode had a maximum power density of 23.6 m W/m^2^, which is 2.3 times higher than carbon cloth.

For improved MFC performance, Zou et al. [63] designed a unique nano-pore structure of activated carbon fibers using bacterial cellulose. By pyrolysis at 600–1000 °C under an argon atmosphere, bacterial cellulose was employed as a raw material to fabricate various surface areas and pore structures of GAC. To provide greater space for bacteria to grow, the carbon fibers were ground into powders and then coated on carbon cloth as an anode. Because of its nanopore structure in favor of strong direct electrochemistry to enhance the ETR, an anode with a pyrolytic temperature of 1000 °C showed the best performance in terms of electricity generation of more than 3.9 A/m^2^ when compared to other pyrolysis temperatures and carbon cloth. Gajda et al. [64] discovered that adding a carbon veil to GAC improved its applicability and robustness in long-term operation significantly. The maximum power density was 21.1 m W/m^2^, representing a 77% increase. Moreover, it is both safe and environmentally friendly.

##### Carbon Nanofibers (CNF)

CNF may have tremendous potential for development as anodes now that electrospinning technology has matured. The electronic conductivity and connectivity of fiber aggregates would be considerably increased after treatment by peroxidation and carbonization procedures [65], and 3D carbon fiber electrodes manufactured by electrospinning have demonstrated outstanding performance for MFC systems. Furthermore, CNF have good long-term stability in solutions. Cai et al. [56] used electrospinning to create a multiwalled nanotubes/CNF composite electrode, showing good conductivity, biocompatibility, and electro-catalytic activity. In the MFC system, the prepared composite anode gives a high-power density of 36,220 m W/m^2^, which is more than the carbon felt anode (16,320 m W/m^2^) [66].

#### 2.3.2. Metal Nanomaterials

Various noble metals and metal materials are used to improve carbon materials to increase conductivity and electro-catalytic activity. Metal materials have a lot better conductivity than carbon materials. However, they are not as extensively used as carbon materials due to their readily corrosive nature. Nanostructure metal materials with excellent catalytic activity, conductivity, and biocompatibility are now being used as anodes in MFC. The nanoparticles used to improve the anode serve as a bridge for electron transfer between the bacteria and the anode. Due to the availability of active sites for bacterial growth on the anode, this leads to an increase in the rates of electricity generation [67].

##### Manganese Dioxide (MnO_2_) and Iron Oxide Based Nanomaterials

Manganese dioxide (MnO_2_) and ruthenium dioxide nanoparticles have good qualities that allow them to lower the internal resistance of the modified electrode and speed up the ETR, although MnO_2_ has poor electronic conductivity. Furthermore, MnO_2_ nanoparticles placed on a traditional carbon anode are non-toxic and low-cost [68].

The anodic surface of electro-active sites can be improved by iron oxide nanoparticles, and they have a magnetic attraction for loading electrochemically active bacteria, allowing for a faster extracellular electron transfer rate. When decomposing pharmaceutically active substances in MFC, carbon cloth coated with Fe_3_O_4_ nanoparticles as anode enhanced a high abundance of Geobacter bacteria. The highest power density attained was 728 m W/m^2^, which was higher than the carbon cloth (680 m W/m^2^). With the engagement of Geobacter, Fe^2+^ and Fe^3+^ can function as electron shuttles at the interface between bacteria and anode for Fe_3_O_4_ nanoparticles [69]. Nakamura et al. [69] discovered that adding nano- Fe_2_O_3_ colloids to MFCs caused S. lochia PV^−4^ bacteria to cross-link with one another, forming a network structure that was helpful to long-distance electron transmission and boosted productiveness by 300%.

To increase the power density of MFCs, composite metal nanoparticles have recently been produced. Using an Amaranthus-mediated technique, copper and iron oxide composite nanoparticles were coated onto the surface of carbon paper to create an effective anode [70]. When employed for dairy wastewater treatment, the maximum power density of 161.5 m W/m^2^ was attained, surpassing pure carbon paper (123.5 m W/m^2^). The low-cost Cu-doped Fe_2_O_4_ nanoparticles improved power generation performance and displayed high stability on carbon paper. 

Through a simple coprecipitation reaction and calcination method, Zeng et al. [71] synthesized a novel bicomponent composite of porous self-assembled nanostructured Ni_0.1_Mn_0.9_O_1.45_ as anode electrocatalyst for power output increase. The electrocatalyst had a porous nanostructure with an ellipsoidal shape, with each ellipsoidal made up of many layered nano building blocks. The specific structure had a large surface area for bacteria to grow on and demonstrated outstanding electrocatalytic activity in allowing extracellular electron transfer between the anode and the bacterium. During long-term operation, the prepared anode in MFC supplied a constant voltage of more than 680 mV, whereas carbon felt as anode only achieved a modest energy output. The form of nanomaterials has been shown to have a significant impact on the extracellular electron transfer rate [72].

##### Titanium Oxide (TiO_2_) and Tin Oxide (SnO_2_) Nanomaterials

**Figure 4:**
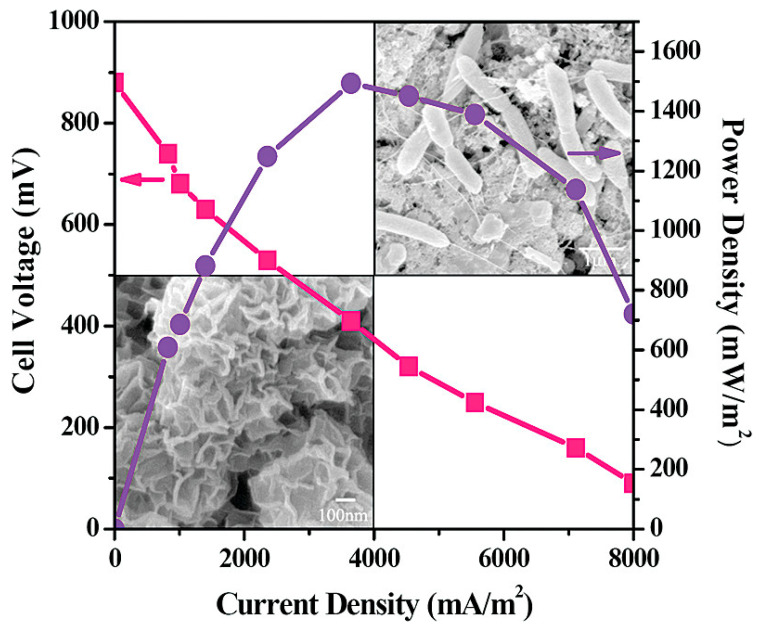
Nanostructured polyaniline (PANI) mesoporous TiO_2_ composite as an anode in *Escherichia Coli* microbial fuel cells (MFCs). Reprinted with permission from [74], Qiao, Y.; Bao, S.J.; Li, C.M.; Cui, X.Q.; Lu, Z.S.; Guo, J. Nanostructured polyaniline/titanium dioxide composite anode for microbial fuel cells. *ACS Nano* **2008**, *2*, 113–119. https://doi.org/10.1021/nn700102s. Copyright @ American Chemical Society (2022).

Tang et al. [73] used TiO_2_ nanoparticles and egg white protein-derived carbon-assembled core-shell nanoparticles to create a nanostructured capacitive layer as anode. When compared to conventional graphite and sponge materials, the novel nanostructured anode demonstrated superior electricity production. The novel 3D anode had a maximum power density of 2.59 (0.12) m W/m^2^, which was 201% and 63% higher than the control group. Improved electrochemical activity and synergetic effects with TiO_2_ and egg white protein-derived carbon composite nanomaterials, such as high specific surface area, excellent biocompatibility, and favorable surface functionalization for the extracellular electron transfer process, were attributed to the increased power output. Qiao et al. have demonstrated the synthesis of unique nanostructured polyaniline (PANI) mesoporous TiO_2_ composite and utilized the prepared material as an anode in *Escherichia Coli* microbial fuel cells (MFCs) [74]. Interestingly, when utilized, the prepared composite anode material exhibited excellent power output (1495 mW/m^2^) in *Escherichia Coli* microbial fuel cells (MFCs) (Figure 4).

**Figure 5:**
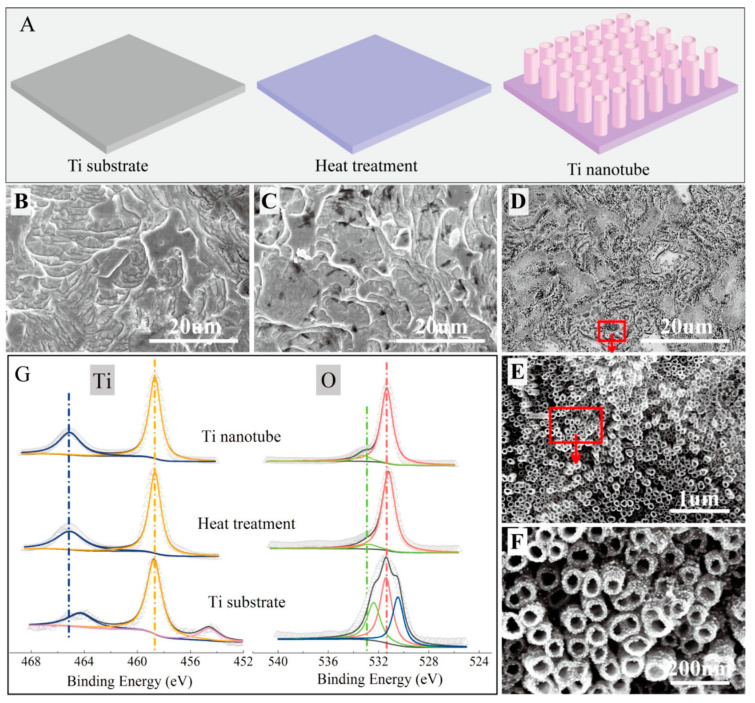
(**A**–**D**) Schematic illustration and SEM images for the Ti substrate after different treatments: (**E**,**F**) SEM images for the Ti nanotube. (**G**) XPS analysis of the prepared materials. Reprinted with permission from [75], Feng, H.; Liang, Y.; Guo, K.; Chen, W.; Shen, D.; Huang, L.; Zhou, Y.; Wang, M.; Long, Y. TiO_2_ nanotube arrays modified titanium: A stable, scalable, and cost-effective bioanode for microbial fuel cells. *Environ. Sci. Technol. Lett.* **2016**, *3*, 420–424. https://doi.org/10.1021/acs.estlett.6b00410. Copyright @ American Chemical Society (2022).

According to Feng et al., in situ growth of titanium dioxide nanotubes (TNs) on a titanium electrode’s surface is an effective technique to transform it into a high-performance anode for microbial fuel cells [75]. The titanium surface was made rougher, more hydrophilic, and better suited for anodic biofilm growth after titanium nanotube modification. Figure 5 exhibits the typical morphologies and fabrication process of Ti nanotubes [75].

**Figure 6:**
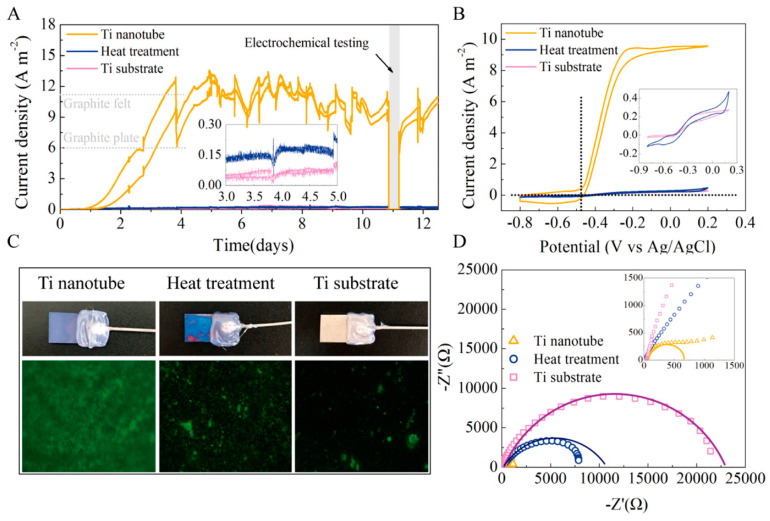
(**A**–**D**) Current output over time of MFCs with different anodes Reprinted with permission from [75], Feng, H.; Liang, Y.; Guo, K.; Chen, W.; Shen, D.; Huang, L.; Zhou, Y.; Wang, M.; Long, Y. TiO_2_ nanotube arrays modified titanium: A stable, scalable, and cost-effective bioanode for microbial fuel cells. *Environ. Sci. Technol. Lett.* **2016**, *3*, 420–424. https://doi.org/10.1021/acs.estlett.6b00410. Copyright @ American Chemical Society (2022).

The maximum current density on this titanium electrode treated with TN was 12.7 A m^−2^, which was 190 times greater than the maximum current density on the unmodified titanium electrode and even higher than the maximum current density on the most widely used carbon felt electrode (Figure 6) [75].

Metal or metal oxide nanomaterials are unable to efficiently construct a 3D structure on the interface of traditional carbon materials to withstand the reduction in mass transfer caused by thick films. Due to a lack of nutrients, the inner layer of bacteria may develop a dead core, resulting in non-conductive detritus on the anode surface [76]. Furthermore, the cytotoxicity of metal or metal oxide nanomaterials must be investigated.

Mehdinia et al. [77] employed reduced graphene oxide (GO) as an anode in an MFC with SnO_2_ nanoparticles on the surface. They achieved a power density nearly five times higher than that using RGO alone. The explanation for this was that the RGO/SnO_2_ material with high conductivity and large surface area significantly aided bacterial biofilm formation and electron transfer.

##### Gold Nanoparticles (Au NPs)

To increase the performance of MFCs, Xiayuan Wu et al. [78] modified carbon cloth anodes with biogenic Au NPs and nanohybrids of multi-walled carbon nanotubes (MWCNTs) mixed with biogenic Au NPs (Au NPs/MWCNT). The results showed that adding Au NPs to MFCs considerably increased their ability to generate power. In particular, Bio-Au/MWCNT nanohybrids as the modifier displayed a good performance. The MFC with the Au NPs/MWCNT electrode exhibited the quickest start-up time (6.74 d) and the highest power density (178.34 m W/m^2^), which were 141.69% and 56.11% shorter and higher, respectively, than the unmodified control. These enhancements were ascribed to the Au NPs/MWCNT nanohybrids on the electrode’s outstanding electro-catalytic activity and great affinity for exoelectrogens. After anode modification, the relative abundance of electroactive bacteria in the biofilm community rose, particularly from the classes Gamma-proteobacteria and Negativicutes, according to high throughput sequencing study.

Au NPs are regarded as appropriate for anode modification in MFCs due to their wide range of beneficial qualities, such as good biocompatibility, high surface-to-volume ratio, and increased conductivity. This is compared to other low-cost metal nanoparticles, such as Ag and Cu, which have proven to show bacterial inactivation and toxicity in certain conditions [79].

### 2.4. Polymer (Polyaniline-Pyrrole) Nanomaterials

Researchers are interested in exploring the potential of conductive polymers with high electrical conductivity and durability as nanostructure materials to improve the energy generation of MFC. The fact that most polymers are insulators is common knowledge. Polyaniline and pyrrole are two commonly discussed conductive polymers. Polyaniline has unique qualities as a conjugated conducting polymer, including good electronic conductivity, good environmental stability, and cheap cost. Additionally, the conductive polymer materials can be easily doped with other nanomaterials to make nanocomposites. The formed nano-composite increases electron transfer and reduces Ohmic resistance as compared to traditional carbon materials. Furthermore, because polyaniline has a positive charge, it attracts electrochemically active bacteria, which increases the power generation of MFC [80].

Polyaniline and CNTs were used to modify the 3D porous sponge in situ to prepare the anode. The prepared bio-anode produced a maximum power output of 4.2 m W/m^2^, which was three times higher than those achieved with a sponge as an anode in MFCs. The high conductivity of carbon nanotubes and polyaniline is critical for increasing power output. Additional contacts for bacterial adhesion and growth are provided by the porous structure and particular surface area. As a result, combining polyaniline and CNTs nanocomposite as an anode modifier has the potential to improve MFC performance [81].

Liu et al. [82] modified carbon cloth with 3,4-ethylenedioxythiophene to increase power output. The power output of 140 m W/m^2^ was achieved, which was increased by 43% compared with the plain carbon cloth as anode in MFCs. The electrochemical investigation demonstrated that coating carbon cloth with 3,4-ethylenedioxythiophene boosted redox active sites and successfully lowered interfacial charge transfer resistance. With Escherichia coli as the biocatalyst, a nanocomposite incorporating carbon nanotubes and polyaniline as an anode modifier in MFC may achieve a maximum power output of 42 m W/m^2^. Furthermore, the composite anode with nanostructure demonstrated high mechanical strength and was environmentally friendly; nevertheless, the long-term operating durability must be explored further. Due to their low cost, conductive polymers as anode modification materials will have a wide range of commercial applications. It is expected to be an effective alternative for noble metal based on the benefits of nano-conductive polymer for anode modification. Meanwhile, the development of new types of conductive polymers and their use in MFC will increase the value of conductive polymers in research. Table 1 summarizes some of the nanomaterials that are used as the anode.

## 3. The Cathode

### 3.1. Materials Used in Cathodes

Like the anode, the cathode material is an essential component of the operation of the MFC to produce electric power or wastewater treatments. The materials used in the cathode must be efficient, stable, and inexpensive [83].

#### 3.1.1. Carbon Materials

Carbon black (CB) is commonly used as a support material for other ORR catalysts because of its high surface area, good electron conductivity, economic feasibility, and high stability. Meanwhile, CB might make itself a metal-free ORR catalyst by chemically modifying or adding functional groups to existing catalytic sites [84]. For example, CB could be modified by polypyrrole to achieve an improved ORR performance by dispersion and coating processes. The ORR potential of polypyrrole/CB cathode changed by roughly 260 mV towards positive potential as compared to pure CB cathode indicating enhanced catalytic activity. Furthermore, a maximum energy density of 401.8 m W/m^2^ was obtained from MFC. The current density of the polypyrrole/CB cathode was much higher than that of 90.9 m W/m^2^ with a pristine CB cathode. Additionally, the power output per cost of the polypyrrole/CB cathode was 15 times higher than that of the Pt cathode [85].

CB cathode could also be modified by other kinds of cathode materials such as iron phthalo cyanine (Fe Pc) that was applied for improvement of CB cathode in MFC using *Enterobacter cloacae* as exoelectrogens. A power output of 400% higher than that of the CB cathode was achieved. When compared to glassy carbon cathode, the electrical analysis revealed that CB/FePc composites had a comparable lower ORR over potential (606 mV) and lower charge-transfer resistance [86]. Although CB has a lower catalytic performance than other cathode materials, the economic cost normalized by produced power is incredible and might be a benefit for future scalable MFC applications. GAC has a high surface area but is not considered a good conductor due to its defective or improper structure. It has been widely used as an inexpensive cathode material in MFCs, and the synthesis and modification of materials used as a cathode have been extensively studied to improve the performance of the catalytic ability of the cathode material [87].

GAC are synthesized by thermal or chemical methods from a wide range of carbon sources. The different methods or materials might provide GAC with differing chemical functional groups, active sites, and surface area, which could have an influence on their catalytic activity [84]. The low catalytic ability of GAC towards ORR limits its use as a cathode in MFCs, so a number of attempts have been made to modify GAC to obtain better catalytic ability. Such as the method of modifying GAC with CB using the sintered method to increase the electrical conductivity and reduce the resistance. This resulted in an increased maximum power density of 90% compared to the unmodified GAC material [88]. In another study, graphene oxide (GO) with activated carbon (AC) was used as another method to modify the GAC, which led to better results than obtained in the case of using CB/GAC due to the good electrical conductivity, which led to higher and better catalytic performance (5 times higher than CB) [89].

#### 3.1.2. Graphite–Graphene Materials

Graphite is a very suitable material as a cathode because of its good electrical conductivity, low price, and good biocompatibility, but it is flawed because its surface area is low and the catalytic or active sites of graphite are very few, which affects the use of graphite as a base material as a cathode in MFC. There are different types of graphite that are manufactured and used as a cathode in MFC. Each type has different properties in terms of electrical conductivity, biocompatibility, catalytic sites, and surface areas such as graphite granules, graphite fiber brush, and graphite brush [90]. For example, pure graphite granules were used as a cathode in MFC, and an electrical power output of 21 W/m^2^ was obtained [91]. In another study, a graphite fiber was used as a cathode in MFC, and two times higher than electrical power output was obtained from the cathode of pure graphite granules due to an increase in the availability of ORR catalytic sites in the graphite fiber cathode than that of the graphite granules cathode [90].

Graphene, the 2D form of graphite, has good properties that qualify it to be used as a cathode electrode material in MFC, such as large surface area, good electrical conductivity, and good biocompatibility in addition to low cost as a raw material for electrodes [92].

#### 3.1.3. Metal-Metal Oxides Materials

Platinum (Pt) is a metal commonly used as a cathode in MFC cells and shows ORR catalytic activity. Platinum is characterized as a good electrical conductor with low resistance, high active surface area, and high electrical energy density when used as a cathode in MFC. However, there is very little interest in using platinum as a cathode due to its high cost in addition to its toxicity to bacteria used in the operation of MFC [93]. The search for alternative materials for platinum as a cathode depends largely on its electrical conductivity, the presence of catalytic active sites, and its high surface area, to have good physical and chemical properties of ORR and low costs, in addition to being non-toxic and environmentally friendly. Examples of metals and their oxides that have been applied as cathodes in MFC are manganese (Mn), iron (Fe), cobalt (Co), and nickel (Ni) [94].

For example, manganese oxides (MnOx) and especially MnO_2_ have been successfully used as a cathode for MFCs, as a catalyst for long-term ORR activity, and as a cost-effective material. MnO_2_ had a clear effect on ORR activity through particle size, polymorphism, and surface area. However, manganese oxides are not used as active cathode materials in MFC because of their poor electrical conductivity and poor mechanical stability of manganese oxide [95]. Another transition metal that has received a lot of attention for its application as effective cathode materials in MFC is copper and vanadium since these metals are inexpensive, widely available, and chemically stable. Vanadium pent oxide (V_2_O_5_) is suitable for practical application as a cathode material in MFC because it is characterized by high surface area, good electrical conductivity, lower electrical resistance, good chemical stability, and low cost [96].

### 3.2. Nanomaterials as Cathode in MFC

Cathode reactions or ORR have two different pathways. The first is a 4-electron path, and the second is a 2-electron path (peroxide path). In the first reaction (4-electron), oxygen is reduced directly to water, where a 2-electron path is transferred, which is the dominant pathway in the noble metal electrodes that are used as catalysts in cathode reactions or ORR. In the second reaction (2-electron or peroxide reaction), hydrogen peroxide is produced, and this reaction is the most common in electrodes that are made of carbon materials, but the 4-electron reaction is preferred over the 2-electron reaction because it reduces oxygen directly to water. Both reactions in MFC depend on pH conditions [6].

In the acidic conditions of the reaction, the peroxide is the primary product in the 2-electron reactions pathways, and H_2_O is the final product in the 4-electron reaction pathway. As for the reaction conditions with a neutral pH, the OH^−^ produced from the reaction accumulates in the catalytic sites on the cathode, which causes a decrease in the kinetic performance [26].

A large number of materials and minerals that are characterized by nanostructures, which at the same time can be used as a cathode in MFCs, have been studied. Among these materials are carbon materials, metals, and metal oxides. The most famous carbon materials whose nanoparticles were used in the manufacture of cathode material are CNTs [6,26]. CNTs are widely used in the manufacture of electrodes in general [97]. It was found that the CNTs have catalytic active sites suitable for cathode reactions or ORR. CNTs were used individually in the manufacture of the cathode material. It was also used in the presence of additional materials involved in the manufacture of the cathode. For example, it was used in the presence of Pt to reduce the amount of platinum used due to the high costs. CNTs contribute to the manufacture of the cathode to reduce the required number of noble catalysts and expensive metals. The results showed that the cathode consisting of Pt/CNTs increased the amount of power output up to 32% compared to using Pt alone. The goal of using Pt/CNTs in the cathode industry is to increase the efficiency of the cathode by increasing the surface area of the electrode and increasing the catalytic sites for the interaction and thus ORR and the consequent increase in the efficiency of the MFC [98].

Silver (Ag) is one of the noble nanomaterials used in the manufacture of the MFC cathode. Silver nanoparticles (Ag NPs) were used to coat the graphite cathode to study its effect on ORR and compare its performance with that of graphite cathode coated with platinum nanoparticles (Pt NPs). The results showed that the performance of the cathodes coated with Ag NPs gave the highest electric current (0.12 mA) compared to the cathodes coated with Pt NPs, which gave 0.04 mA. However, Ag NPs are blamed for the fact that they inhibit or prevent the growth of biofilms on the cathode, in addition to their high cost. This hinders the use of Ag NPs from being used as a suitable cathode to perform ORR and thus affects the improvement of the efficiency of MFC [99].

Precious metal catalysts have been shown to perform well in ORR, but their high cost and rarity in nature impose restrictions on their use. As for the transition metal catalysts, they were of great importance in their application in bio-electrochemical systems and thus their use in MFC. Cobalt (Co) catalysts showed excellent results in ORR, and their use as a cathode in MFC led to an increase in the rate of ORR. For example, cobalt tetra-methyl phenyl porphyrin (CoTMPP) was tested in MFCs with a single-chamber cathode antenna. Similar results were obtained for using Pt as a cathode in MFC [100]. The use of additives to cobalt, such as adding cobalt oxide (CoO_2_) to Fe Pc, was also tested by Ahmed et al. [101]; the results showed that large electrical energy was produced, about 654.32 mW/m^2^ compared to using Co alone. In a study by Yuan et al. [102], Fe Pc was used as a catalyst for ORR by adding it to each of the amino-functionalized multi-walled platinum nanotubes to build the MFC cathodes. The results showed that the use of the Fe Pc compound led to an increase in the ORR and, thus, an increase in power output. Additionally, these materials improved the performance of platinum cathodes, as the results showed that a power output obtained reached 601 m W/m^2^.

Manganese (Mn) is one of the metal catalysts that have received great interest in the past years due to its low cost and availability in nature. Manganese oxide nanoparticles (MnO_2_NPs) were used, which were prepared in a relatively simple way, but gave good results in ORR. Hybrid MnO_2_NPs were prepared by Haoran et al. [103] by a hydrothermal method as a cathode in the MFC, which led to an increase in the ORR, and the reaction was carried out via the 4-electron pathway. MnO_2_NPs were also added to graphene as cathode materials, and the results showed a higher energy density compared to the cathodes that were made of MnO_2_ only or graphene only.

**Figure 7:**
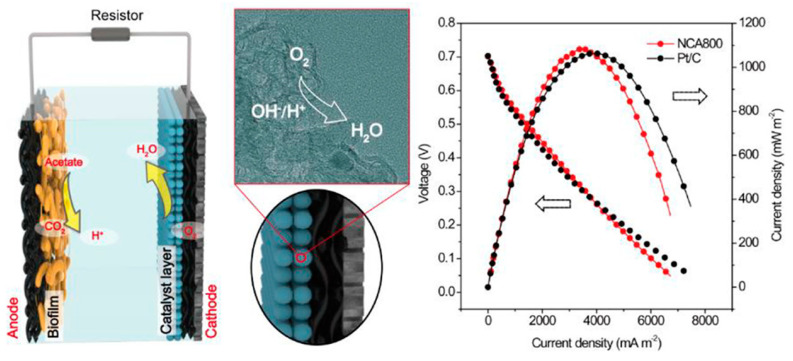
Utilization of nitrogen-doped carbon aerogels for microbial fuel cell. Reproduced with permission from [106].

Spinel oxides are nano-oxides that have been used as catalysts. The general formula for spinel oxide is AB_2_O_4_, where A and B are metal cations. The type of cations depends on their composition, including normal, inverse, tetrahedral and cubic structures, where the cations are placed according to their type in tetrahedral or octahedral sites. The results showed that the cubic structure of spinel oxides gave a higher efficiency in ORR compared to the tetragonal-structured spinel oxide. Spinel oxides that have used Co cations have received great attention due to their low cost and multiple Co valences. CoO_2_ and MnO_2_ were used in MnxCul-xCo_2_O_4_ spinel oxides to raise their efficiency. These compounds were prepared by a relatively simple hydrothermal method and added to carbon compounds used as cathodes in MFC, and their performance was compared to Pt used as cathodes in power output density and chemical oxygen demand (COD) removal. The results showed that the use of MnxCul-xCo_2_O_4_ compounds gave a high-power output density of about 570 m W/m^2^, an increase of about 87% of the energy generated by Pt. The results also showed that with the use of MnxCul-xCo_2_O_4_ compounds in the cathode compositions, about 56% of the COD was removed after about 240 h of operation of the MFC [104,105]. Using nitrogen-doped carbon aerogels prepared by pyrolysis of polyacrylonitrile at controlled temperatures, Yang et al. reported the fabrication of a microbial fuel cell [106]. The fabricated MFC based on the aerogel exhibited a maximum power density of 1048 ± 47 mW.m^−1^ and a mass-specific power of 52.4 mW.g^−1^ (Figure 7). Table 2 summarizes some of the nano-materials that are used as the cathode.

## 4. The Membrane

MFC performance is heavily reliant on PEM. Transporting protons from the anode to the cathode selectively while preventing both substrate transfers from the anode to the cathode and oxygen crossover from the cathode to the anode [6]. PEM are widely used in commercial microbial fuel cells (MFC), such as Nafion and Ultrex, but their use is limited by their high cost and disadvantages, such as substrate and oxygen transfer, biofouling, and cation transport. Therefore, due to these shortcomings, the trend has been to manufacture alternative materials based on nanoparticles, such as polymeric/inorganic, because of their applications in energy production. Moreover, the presence of nanoparticles in the composition of PEM led to an improvement in the chemical and physical properties of the membrane, such as impairing the permeability of the protons and the thermal and mechanical stability of the membrane [107].

### 4.1. Nanomaterials as Membrane in MFC

#### 4.1.1. Poly Ether Sulfone (PES)/Fe_3_O_4_ as Nano-Membrane

PES is one of the most important polymers that have been used as nanoparticle-type membrane support materials. Rahimnejad et al. [108] manufactured a new type of nanocomposites membranes based on the use of Fe_3_O_4_ nanoparticles and focused on the effect of different amounts of nanoparticles/polymer on the performance of MFC using *Saccharomyces cerevisiae* as a biocatalyst, glucose as a substrate and neutral red as a mediator at the anode. Using the casting method, iron-based nanoparticles were synthesized using a solution of FeCl_2_ andFeCl_3_ and sodium hydroxide as precursors. The membranes were fabricated containing Fe_3_O_4_ particles at concentrations of 10, 15, and 20 wt% of Fe_3_O_4_. The results showed that the increase in the pore size increases with the increase in the number of nanoparticles of Fe_3_O_4_. However, the results showed that in the membranes containing concentrations of 15 and 20% of Fe_3_O_4_ nanoparticles, the pore size was not uniform, and the surface roughness was very high due to the appearance of some agglomerates. It was also found that membranes containing 15% of Fe_3_O_4_ nanoparticles gave an increase in the power output by 29% over the value obtained using Nafion 117 (15.4 m W/m^2^). Membranes containing 20% of nanoparticles gave lower values of power output than those containing 10% of nanoparticles. The results also show that iron nanoparticles have good properties such as conductivity, magnetism, or high catalytic activity, which clearly favors the performance of MFCs.

In another related study, melt extrusion technology was used to fabricate Fe_3_O_4_ nano-membranes using PES. Di Palma et al. [109] prepared Fe_3_O_4_ nanoparticles by co-precipitation of Fe^+3^ and Fe^+2^ using NH_4_OH as a precipitating agent. To obtain composite nano-sheets of PES and two different concentrations of magnetite nanoparticles, 5% and 20%, these membranes were used and tested in MFC containing sodium acetate as a carbon source. The results of this work showed agreement with the results obtained by Rahimnejad et al. [108] of increasing the roughness of the nanoparticles with an increase in the number of nanoparticles as well. Membranes containing a concentration of 20% Fe_3_O_4_ gave a maximum power output of 9.59 mW/m^2^ with a value similar to the power output using the CMI-7000 commercial membrane, which is 12.58 m W/m^2^. In MFC, the results also showed that increasing the concentration of magnetite nanoparticles in the membranes increases the performance of MFC in terms of electrical energy production. The membranes containing concentrations of magnetite nanoparticles were tested in wastewater treatment using MFC. The results showed that this type of membranes removes total organic carbon (TOC) by 75% and a columbic efficiency by 11.36%. From these results, it is clear that the membranes containing 20% magnetite nanoparticles show a balance between chemical and electrochemical performance, and therefore they are very suitable in the operation of MFC in the cases of energy production and wastewater treatment.

#### 4.1.2. Sulfonated Poly Ether Ketone (SPEK) as Nano-Membrane

SPEK is one of the types of polymers that are widely used in preparing membranes of Fe_3_O_4_ nanoparticles, as reported by Prabhu and Sangeetha [110]. First, a sulfonated polymer was obtained by mixing sulfuric acid and SPEK. Second, by casting method, polymer membranes were prepared by dissolving an appropriate amount of SPEK in N-methyl pyrrolidone. Finally, Fe_3_O_4_ nanoparticles were prepared from FeCl_3_ and FeCl_2_ and then added to the casting solution. Membranes with different concentrations of magnetite nanoparticles of 2.5, 5, 7.5, and 10% were used to study the effect of these concentrations on the properties of the membrane and thus their effect on the performance of MFC using *E. coli* (DH5-α) as active bacteria at the anode and glucose as a carbon source and platinum-loaded carbon cloth as a catalytic cathode for the oxygen reduction reaction. The results showed that increasing the concentration of magnetite nanoparticles increased the roughness of the membranes. Concentrations of 2.5, 5, and 7.5% of magnetite nanoparticles in the membranes led to a decrease in the oxygen transport rate. A concentration of 10% of magnetite nanoparticles in the membranes led to an increase in the oxygen transfer rates due to the space in the internal structure of the membrane. The maximum power output reached 104 mW/m^2^ for SPEK membranes containing 7.5% magnetite nanoparticles, which is higher than the power output when using the commercial Nafion 117 membrane and also higher than the power output when using SPEK membranes that do not contain magnetite nanoparticles. The membranes containing 7.5% magnetite nanoparticles gave a coulomb efficiency of 87%, and the surface roughness of these membranes prevented the transfer of oxygen from the cathode to the anode, which increased the efficiency of the MFC performance. However, the membranes containing 10% magnetite nanoparticles and the increase in the roughness of the membranes had a negative effect on the efficiency and performance of the MFC. The membranes containing concentrations of magnetite nanoparticles showed lower selectivity in transporting cations such as sodium, calcium, potassium, magnesium, and ammonium than the commercial Nafion membranes, which favored the selectivity of protons. These results showed that SPEK membranes containing Fe_3_O_4_ nanoparticles are a suitable alternative to increase the efficiency of MFC than commercial membranes.

Membranes made of SPEK have been doped with montmorillonite nanoparticles (MNPs) in addition to magnetite nanoparticles. Hasani-Sadrabadi et al. [111] investigated the impact of the sulfonation degree of SPEK and the amount of MNPs on single-chamber MFC performance. This sample was water soluble while having a maximal ion exchange capacity of 89% sulfonation degree. The maximum sulfonation degree that permits the sample to remain mechanically and hydrolytically stable was determined to be 82%. In terms of liquid uptake and proton conductivity, both parameters increase as the sulfonation degree increases. However, because the sulfonated group may impact the crystallinity of the SPEK structure, the larger the number of sulfonate groups, the higher the oxygen transfer rate. The oxygen molecules can permeate through formed ionomeric nanochannels, which increase as the sulfonated group increases. Nonetheless, the oxygen permeability of the samples with varying degrees of sulfonation is lower than that of Nafion 117. The selectivity of the membrane improves with the increase in the number of sulfonated groups, reaching a maximum sulfonation degree of 70%. As a result, this was the polymeric matrix used for the fabrication of MNPs membranes. Membranes containing 3% nanoclay had the maximum selectivity. As a result, SPEK-based membranes with a sulfonated degree of 70% and a nanoclay content of 3% were evaluated as separators in single-chamber MFCs. In this case, E. coli Top10F was used as bacteria in the anodic chamber, glucose as the carbon source, carbon cloth as anode, and a carbon black blend of platinum as a cathode. The results demonstrate that the nanocomposite membrane, which contains 70% SPEK and 3% nanoclay, allows MFCs to produce around 40% more power than the commercial Nafion 117 membrane. SPEK-based nanoclay membranes are viable alternatives to commercial MFC separators because of their high-power output, cheap cost materials, simple synthesis process, and low oxygen transfer.

In order to improve the characteristics of SPEK membranes, sulfonated silica has also been employed as a nanomaterial. Sivasankaran and Sangeetha [112] investigated the effect of adding SiO_2_ and sulfonated SiO_2_ nanoparticles on SPEK-type membranes on proton conductivity and separator performance in MFCs. They experimented with various concentrations of sulfonated silica in the SPEK matrix (2.5, 5, 7.5, and 10%), comparing the results to those obtained with SPEK/SiO_2_-type membranes and Nafion 115. Before being employed as a separator in a single chamber air cathode MFC supplied with domestic wastewater in batch mode, the membranes were characterized. Electrochemical impedance spectroscopy (EIS) generated Nyquist plots revealed that membrane resistance reduces as the concentration of sulfonated silica rises, with the lowest resistance seen in membranes containing 7.5%. This might be due to the low conductivity of SiO_2_ particles, which increase membrane resistance while lowering conductivity. However, the addition of a more conductive compound, such as SiO_2_-SO_3_H, lowers the membrane’s resistance and hence improves its conductivity. Sulfonated silica membranes had higher conductivity than SPEK-SiO_2_ and SPEK membranes, with the highest conductivity seen in membranes containing 7.5% of SiO_2_-SO_3_H (1.018 Scm^−1^). Sulfonated silica concentrations above 7.5% increased membrane resistances due to agglomeration of the sulfonated silica over the SPEK matrix, reducing conductivity. In terms of MFC separators, membranes made with 7.5% of SiO_2_-SO_3_H allow MFC to reach the maximum power output (1008 mW/m^2^), which is higher than Nafion 115 (320 mW/m^2^). The columbic efficiency is around 90%, which is higher than the efficiency of the other materials examined. The addition of SiO_2_-SO_3_H to SPEK-based membranes increases conductivity and so improves their efficiency as MFC separators, according to this study. Membranes containing 7.5% of SiO_2_-SO_3_H have similar conductivity and water uptake to commercial membranes such as Nafion 115. However, oxygen mass transfer is an order of magnitude lower for sulfonated silica SPEK membranes, which improves the power performance of MFCs.

#### 4.1.3. Polyvinylidene Fluoride (PVDF) as Nano-Membrane

Because of its thermal and chemical stability, as well as its biocompatibility, PVDF is extensively utilized as a polymer matrix for many types of membranes. This material has demonstrated promise in a wide range of research disciplines, including separation processes, bioenergy generation in direct methanol fuel cells, and biological applications. With all these advantages, Shahgaldi et al. [113] used the electrospinning process to produce PVDF nanofibers in 2014. Their research aimed to develop PDVF nanofiber/Nafion membranes for use as separators in double chamber MFCs that use *Saccharomyces cerevisiae* as a biocatalyst in the anode and glucose as a carbon source. They also looked at how different combinations of PDVF nanofibers and Nafion (10 wt%/0.2 g, 18 wt%/0.4 g, and 29 wt%/0.6 g) affected MFC performance. The results showed that membranes containing 0.4 g of Nafion allow MFCs to reach their maximum power output (4.9 mW/m^2^), whereas MFCs using membranes containing 0.6 g of Nafion perform similarly to commercial Nafion 117 in terms of power output. The higher proton conductivity of membranes containing 0.4 g of Nafion can explain this. This membrane is also the most efficient in terms of columbic efficiency (12.1%). On the other hand, membranes constructed with a PVDF nanofiber/Nafion proportion of 29 wt%/0.6 g have higher power output and columbic efficiency than membranes containing 10 wt%/0.2 g PVDF nanofiber/Nafion. These results lead them to believe that the amount of PVDF nanofiber in Nafion composite membranes is an important component. In terms of wastewater treatment capacity, all the systems investigated can remove more than 70% of COD, making them all suitable for wastewater treatment.

Rudra et al. [114] studied the production of a membrane consisting of polyvinyl alcohol (PVA) and sulfonated styrene (SS) by adding different concentrations of graphite oxide (GO) (0.2%, 0.4%, and 0.6% by weight) to be used as a separator in MFCs as a polymer matrix for nanocomposites membranes. *Lysinibacillus* sp. was used as active bacteria in the anode, which was fed synthetic wastewater. The hydrophilicity of PVA is lowered by crosslinking it with SS and adding GO to it. In addition, the presence of GO decreases oxygen diffusion across the membranes. In terms of resistance, the Nyquist plots revealed that nano-composite membranes containing 0.4 wt% GO had the lowest resistance (5.4 Ω) and the highest maximum conductivity of all the materials synthesized. In terms of power output, MFCs using this type of nano-composite membrane can produce a maximum power of 194 mW/m^2^, which is higher than the other materials examined. In terms of wastewater treatment capacity, all manufactured materials contain 0.2% of GO (88.97%). Then, 70% of COD was removed after 25 days of MFC operation. The results showed the possibility of using these nanomaterials as low-cost membranes in MFC for energy production and wastewater treatment at the same time.

#### 4.1.4. Sulfonated Polystyrene-Ethylene-Butylene-Polystyrene (SPEBP) as Nano-Membranes

Of the membranes that are used in the MFC, SPEBP was used in the manufacture of the nanocomposite-type membranes. Sivasankaran and Sangeetha [112] added sulfonated TiO_2_ nanoparticles whose concentrations ranged between 2.5 and 10% to the SPEBP matrix. These materials, which were manufactured in MFCs that operate using wastewater, bacteria, and glucose as a carbon source, have been tested. Increasing the content of sulfonated TiO_2_ leads to an increase in the ionic conductivity of the samples with a maximum concentration of 7.5% of titanium nanoparticles (3.35 μL g^−1^). This is due to the increase in the acidic sites resulting from the presence of sulfonated TiO_2,_ and the increase in proton conduction was observed with a maximum concentration of 7.5% of sulfonated TiO_2_ (3.57 × 10^−2^ S. cm^−1^) for the prepared nanocomposites membranes. However, increasing concentrations of titanium nanoparticles lead to a decrease in oxygen transport, which benefits their use in MFCs. In terms of application as an MFC separator, the membranes containing 7.5% of sulfonated TiO_2_ gave a maximum power output of (1345 mW/m^2^), which is four times higher than that of using Nafion 117 and columbic efficiency of (87%).

#### 4.1.5. CNF/Nafion and Activated Carbon Nanofiber (ACNF)/Nafion as Nano-Membranes

Nafion is one of the solutions used in membranes with nanocomposites. Ghassemi et al. [115] studied the use of CNF/Nafion and activated carbon nanofiber (ACNF)/Nafion membranes and compared the use of these membranes with commercial membranes Nafion 112 and Nafion 117 in the performance and operating efficiency of MFC in power generation and wastewater treatment. CNF/Nafion and ACNF/Nafion membranes are characterized by low surface roughness and decreased pore size of the membranes.

Small pore sizes reduce oxygen transport across the membrane, which is very beneficial for the performance of MFC. The new nano-membranes were tested in a double chamber MFC with glucose as a carbon source to feed the bacteria. The results showed that the nano-composites membranes manufactured from ACNF/Nafion gave the highest maximum power output of 57.64 mW/m^2^ and removed about 70% of the COD compared to the results obtained from the use of commercial membranes Nafion 112 and Nafion 117. The results obtained from using nano-membranes CNF/Nafion and ACNF/Nafion are suitable alternatives to commercial membranes used in MFC in power generation and wastewater treatment. Table 3 summarizes some of the nanomaterials that are used as the membrane.

## 5. Conclusions and Future Directions

This review deals with the applicability of nanomaterials in improving the performance and efficiency of MFC in the manufacture of anode and cathode electrodes and proton exchange membranes. CNTs, CNF, graphene, GAC, CNS, and metal oxides such as iron oxide, MnO_2_, gold, silver, cobalt, tin oxide, and spinel oxide are among the most famous nanomaterials that have been used to improve the efficiency of the anode and cathode electrodes by increasing the surface area of the electrodes, increasing the rates of electron transfer, and increasing biocompatibility with microorganisms, biofilm formation, oxidation reactions, and ORR. The addition of nanomaterials to the components of proton exchange membranes, such as Fe_2_O_3_, Fe_2_O_4_, SiO_2_, MnO_2_, GO, TiO_2_, CNF, and ACNF, led to an improvement in the chemical and physical properties and thermal stability of the membranes, thus increasing the transfer rates of protons and preventing the transfer of oxygen from the cathode chamber to the anode chamber compared to the performance of commercial membranes. The use of nanomaterials had a substantial effect in improving the performance and efficiency of MFC in the production of electrical energy and wastewater treatment.

From the above, it becomes clear the importance of using nanomaterials to improve the efficiency of electrodes and membranes used in the operation of MFCs. Therefore, we, the authors of this review, recommend the use of nanomaterials to improve the operating efficiency of microbial fuel cells in the future.

## Figures and Tables

**Table 1:** Nanomaterials used as anode material.

Nanomaterials	Based Materials	Results	Ref.
CNTs + carbon paper	Carbon nanomaterials	Power output: 290 m W/m^2^	[55]
Graphene nanomaterials	Graphene nanomaterials	Power output: 2668 m W/m^2^	[60]
Activated microporous-mesoporous carbon	Carbon nanomaterials	Power output: 23.6 m W/m^2^	[62]
GAC nanomaterials	Graphene nanomaterials	Power output: 21.1 m W/m^2^	[64]
Carbon nanofibers (CNF)	Carbon nanomaterials	Power output: 36,220 m W/m^2^	[56]
Fe_3_O_4_ nanoparticles	Metal nanomaterials	Power output: 728 m W/m^2^	[73]
TiO_2_ nanoparticles	Metal nanomaterials	Power output: 2.59 m W/m^2^	[72]
Au NPs/MWCNT	Metal nanomaterials + Carbon nanomaterials	Power output: 178.34 m W/m^2^	[78]
Polyaniline and CNTs	Carbon nanomaterials	Power output: 42 m W/m^2^	[81].
Modified carbon cloth with 3,4-ethylenedioxythiophene	Carbon nanomaterials	Power output: 140 m W/m^2^	[82]

**Table 2:** Nanomaterials used as cathodes.

Nanomaterials	Based materials	Results	Ref.
Pt/CNTs	Metal/Carbon nanomaterials	Power output up to 32% compared to using Pt alone	[98]
Ag NPs	Metal nanomaterials	Electric current: 0.12 mA	[99]
Pt NPs	Metal nanomaterials	Electric current: 0.04 mA.	[99]
CoO_2_	Metal oxide	Power output: 654.32 mW/m^2^	[101]
Platinum nanotubes	Metal	Power output: 601 m W/m^2^	[102]
MnxCul-xCo_2_O_4_	Spinel oxides (Nano-oxides)	Power output: 570 m W/m^2^ COD removal: 56%	[104,105]

**Table 3:** Nanomaterials used as membranes.

Membrane	Nanomaterials	Results	Ref.
Poly Ether Sulfone (PES)/Fe_3_O_4_ as nanomembrane	Fe_3_O_4_ nanoparticles (15%)	Power output: 15.4 m W/m^2^ Remove a total organic carbon (TOC): 75% Columbic efficiency: 11.36%	[108]
	Fe_3_O_4_ nanoparticles (20%)	Power output: 9.59 mW/m^2^	[108]
Sulfonated Poly Ether ketone (SPEK) as Nano-membrane	Fe_2_O_4_ nanoparticles (10%)	Power output: 104 mW/m^2^	[110]
	Fe_2_O_4_ nanoparticles (7.5%)	Columbic efficiency: 87%	[110]
	SiO_2_-SO_3_H (7.5%)	Power output: 1008 mW/m^2^ Columbic efficiency: 90%	[112]
Polyvinylidene fluoride (PVDF) as Nano-membrane	Nano-fiber/Nafion 10 wt%/0.2 g	COD removal of more than 70%	[113]
	Nano-fiber/Nafion 18 wt%/0.4 g	Columbic efficiency: 12.1%	[113]
	Sulfonated styrene (SS) 0.4 wt/Graphite oxide (GO) 0.2%	Power output: 194 mW/m^2^ COD removal: 70%	[114]
Sulfonated Polystyrene -Ethylene-Butylene-Polystyrene (SPEBP) as Nano-membranes	TiO_2_ nanoparticles (7.5%)	Power output: 1345 mW/m^2^ Columbic efficiency: 87%	[112]
CNF/Nafion and activated carbon Nano-fiber (ACNF)/Nafion as Nano-membranes	Activated carbon Nano-fiber (ACNF)/Nafion	Power output: 57.64 mW/m^2^ COD removal: 70%	[115]

## Data Availability

Not Applicable.

## Abbreviations

2D	Two-dimensional
3D	Three-dimensional
ACNF	Activated carbon nano-fiber
Ag NPs	Silver nanoparticles
Au NPs	Gold nanoparticles
CB	Carbon black
CNF	Carbon nano-fibers
CNTs	Carbon nanotubes
Co	Cobalt
COD	Chemical oxygen demand
CoO_2_	Cobalt oxide
CoTMPP	Cobalt tetra-methyl phenyl porphyrin
EIS	Electrochemical impedance spectroscopy
ETR	Electron transfer rate
Fe Pc	Iron phthalo cyanine
GAC	Granular activated carbon
GO	Graphene oxide
MFCs	Microbial fuel cells
MnO_2_	Manganese dioxide
MnO_2_NPs	Manganese oxide nanoparticles
MNPs	Montmorillonite nanoparticles
MWCNTs	Multi-walled carbon nanotubes
ORR	Oxygen reduction reaction
PEM	Proton exchange membrane
PES	Poly Ether Sulfone
Pt	Platinum
Pt NPs	Platinum nanoparticles
PVA	Polyvinyl alcohol
PVDF	Polyvinylidene fluoride
RGO	Reduced graphene oxide
SnO_2_	Tin oxide
SPEBP	Sulfone polystyrene-ethylene-butylene-polystyrene
SPEEK	Sulfonated Poly Ether ketone
SS	Sulfone styrene
TOC	Total organic carbon
V_2_O_5_	Vanadium pentoxide
